# Designing a Patient Room as a Fall Protection Strategy: The Perspectives of Healthcare Design Experts

**DOI:** 10.3390/ijerph18168769

**Published:** 2021-08-19

**Authors:** Melissa Piatkowski, Ellen Taylor, Bob Wong, Dorothy Taylor, K. Bo Foreman, Andrew Merryweather

**Affiliations:** 1 The Center for Health Design, Concord, CA 94520, USA; mpiatkowski@healthdesign.org; 2 College of Nursing, University of Utah, Salt Lake City, UT 84112, USA; bob.wong@nurs.utah.edu; 3 College of Engineering, University of Utah, Salt Lake City, UT 84112, USA; dorothy.taylor@utah.edu (D.T.); bo.foreman@hsc.utah.edu (K.B.F.); a.merryweather@utah.edu (A.M.); 4 Department of Physical Therapy and Athletic Training, University of Utah, Salt Lake City, UT 84108, USA

**Keywords:** falls, evidence-based design, patient room, frail/elderly, risk, augmented reality

## Abstract

Despite decades of research into patient falls, there is a dearth of evidence about how the design of patient rooms influences falls. Our multi-year study aims to better understand how patient room design can increase stability during ambulation, serving as a fall protection strategy for frail and/or elderly patients. The aim of this portion of the study was to ascertain the architect’s perspective on designing a room to mitigate the risk of falls, as well as to evaluate the face validity of a predictive algorithm to assess risk in room design using the input of a design advisory council (AC). The purpose of this paper is to provide insight into the design process and decision-making for patient rooms; summarize the impressions of industry experts about the configurations and layout of the patient rooms tested in a preliminary augmented reality model; establish the face validity of modeled heat maps depicting risk; and report the results of a pre-meeting and post-meeting survey of expert opinions. Feedback was coded using human factors/ergonomic (HF/E) design principles, and the findings will be used to guide further development of an “optimal” prototype room for human subject testing. The results confirm the challenges that architects face as they balance competing priorities and reveal how a participatory process focusing on preventing falls can shift assumptions about design strategies, especially subtle changes (e.g., toilet orientation).

## 1. Introduction

Falls resulting in serious injury while a patient is being cared for in a healthcare setting have been classified in the United States (US) as a “never event” or “serious reportable event” since 2002 [1]. As a result, care resulting from such a fall is no longer reimbursed by the Centers for Medicare and Medicaid Services (CMS) under both the HAC (Hospital-Acquired Conditions) Reduction Program and Deficit Reduction Act and Hospital-Acquired Conditions (Present on Admission Indicators) [2,3,4]. Despite some improvement, the rate of change in injurious falls continues to be lower than several other HACs. The scorecard data from 2014–2017, for example, indicate a 5% reduction in the rate of injurious falls, as compared to adverse drug event reductions (28%) and reduced rates of *Clostridioides difficile* infections (37%) [5]. Unfortunately, the high incidence rates of injurious falls and understanding their causes is a global phenomenon.

While risks for inpatient falls include intrinsic factors such as cognitive or mobility limitations, acknowledged extrinsic risk factors include physical hazards and latent conditions in the patient room design [6,7,8]. The influence of built environment conditions as a risk factor for falls has been studied using biomechanics around the patient bed [9,10] and bathroom [11], experimental trials of specific material interventions (e.g., flooring) [12,13], and the nascent development of predictive models to evaluate room design undertaken for the present study [14,15]. This paper represents part of a multi-year research initiative to better understand how the patient room can be designed to increase patient stability during ambulation as a fall protection strategy. Using a computerized predictive risk algorithm [15], augmented reality (AR), and a survey, patient room designs were evaluated by gathering the perceptions of architects and regulators (see Table S1).

To best support the capabilities and limitations of patient tasks in patient rooms, it is imperative to establish a baseline understanding about which design principles and features should therefore be prioritized, from multiple points of view. There has been an increasing awareness about the diverse views of stakeholders to inform the design process [16,17,18,19]. While it is certainly necessary to gather empirical data about patients using a prototype design to inform a proactive approach for safety, it is also important to address the assumptions made by those designing the room.

## 2. Materials and Methods

### 2.1. Advisory Council (AC)

In order to advance the development of an evidence-based prototype safe patient room, the research team created a purposive sample of experts in the field of healthcare design. These experts were invited to participate as the National Design Advisory Council (AC) for the study. The AC includes six US-based healthcare design experts representing healthcare organizations, regulatory bodies, professional association knowledge community (KC) leadership, and architecture/design/planning firms (Table 1). A seventh member from the United Kingdom participated in the project virtually.

### 2.2. Room Design

**Figure 1:**
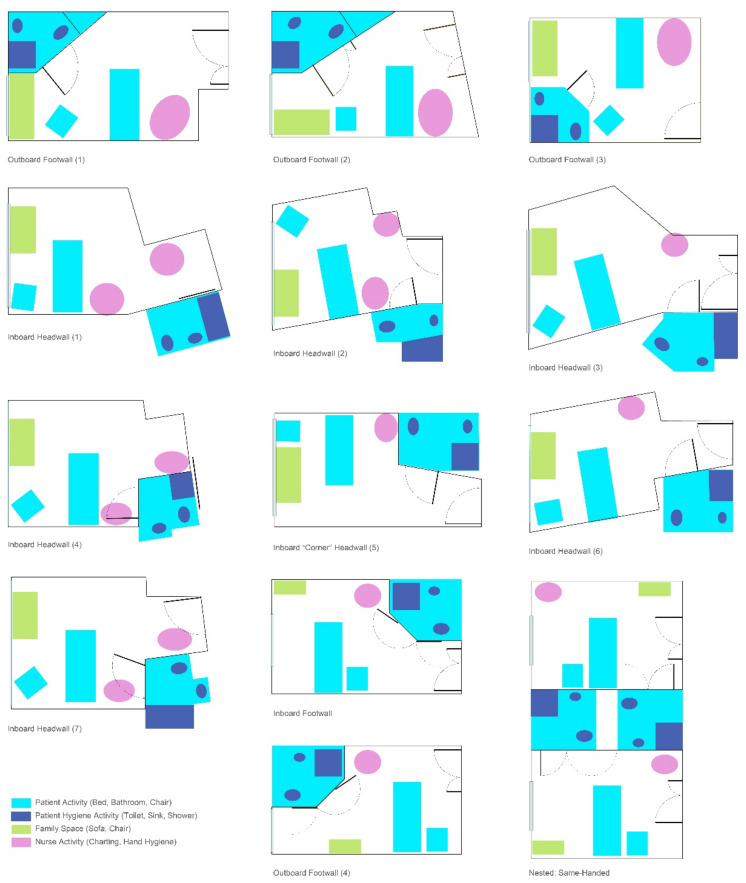
Room configurations submitted by the AC.

As part of their participation, the AC members were asked to share the design of a medical/surgical patient room design from a completed project. Multiple configurations (Figure 1) reflected variations traditionally found, such as inboard (bathroom next to the hallway room entry), outboard (bathroom along the exterior wall), headwall (bathroom on the wall at the head of the patient bed), and footwall (bathroom on the wall at the foot the patient bed). While some layouts had similar configurations, differences were reflected in bathroom door types (i.e., single leaf, double doors, door and relief panel, sliding door, doors with glass), as well as bathroom configurations (e.g., toilet location, grab bar locations/types). Each design was evaluated through two integrated approaches:

1. A computer algorithm of risk [15]; and
2. A “live” AR evaluation session of selected room designs.

### 2.3. AR Approach

The research team selected the AR approach based on the flexible and modular framework and the convivence it provides to quickly transition between environments, reduce build time, and setup and integrate a combination of virtual and physical objects to evaluate human perception and performance. Recent studies in the field of architectural design have shown AR to be an effective way to closely simulate real experiences to evaluate various options quickly without having to make physical modifications, as with a full-scale physical model [20,21,22]. The advantage of AR over virtual reality (VR), is that the technology overlays (or augments) virtual objects onto actual physical objects, allowing users to move around in a real space, touching and interacting with major components of the space (i.e., laying on an actual bed; sitting on an actual toilet) while viewing virtual objects that are not physically represented in the room (e.g., a window, a door to the bathroom, walls). Each room layout was designed with the ability to turn lighting on and off; however, only daytime conditions within the AR were simulated. The AR environment was created using Unity software (San Francisco, CA), and participants were fitted with a Valve Index Head Mounted Display (HMD) and two Valve Index controllers.

### 2.4. Risk Evaluation

**Figure 2:**
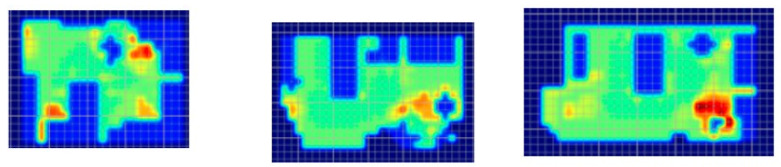
Heat maps depicting risk generated by the computer algorithm. A color gradient of blue representing low risk and red representing high risk was chosen for visualizing risk severity.

In the first phase of the AC engagement, researchers created a preliminary version of risk-based algorithm-modeled “heat maps” of each patient room (Figure 2) provided by the AC. The heat maps reflected a spectrum of “supportive” features in the room (e.g., furniture, walls, lighting), as well as trajectories of motion for common patient ambulation paths [15]. The heat maps did not account for the intrinsic conditions of the patient or features where levels of risk could not be quantified in the literature.

The heat maps were reviewed by each submitting AC member via video conference. During the call, the AC member was asked about whether the models were thought to reasonably represent the risk of falls for the room provided, solely based on their expert stance as a healthcare designer. Most questions and comments were associated with the “missing” elements (e.g., medical equipment, not accurately accounting for high levels of falls leaving the bed due to interactions with intrinsic patient conditions). Though opinions varied regarding some of the assigned levels of risk represented in the heat maps, the AC was excited about the use of this tool to visualize potential risk in a variety of patient room designs.

### 2.5. Gathering In-Person Feedback from the AC

Subsequently, the AC assembled for an in-person meeting at the study site to engage in a further evaluation of a selection of submitted rooms. The meeting was seen as a chance to both gather feedback on room design and assess initial face validity of the risk algorithm. For the purposes of this study, face validity is conceptualized as the following: “Individuals knowledgeable about the system are asked whether the model and/or its behaviour are reasonable. For example, is the logic in the conceptual model correct and are the model’s input–output relationships reasonable? [23]”. The meeting started with the AC completing a survey of Likert-scale and open-ended questions asking their expert opinions about room design to optimize stability (Supplementary File S1). This was followed by research team presentations providing an overview of prior research on patient falls and background of the current study. Following the introduction and pre-session survey:

- The AC panel shared their expertise on how the design process starts, how consensus is built, and how priorities are established.
- The AC panel participated in walking through a set of patient tasks in AR patient rooms.
- An initial debrief included a review of the preliminary heat maps as compared to the AR experience, followed by an overall debrief of the AR experience through a group discussion prompted by probing questions from the research team.
- A more comprehensive debrief of the day included a range of feedback and identified next steps.
- A post-session survey (the same survey from the morning) was administered to evaluate any change in opinions.

After sharing their views on patient room design, the AC was brought to the room where the AR patient tasks were to be completed. The physical room was outfitted with a patient bed, a chair, a toilet, chairs for the observers, and a station for controlling the AR simulation. One of the co-investigators provided instructions to the AC participants about the intended AR activity. Each participant in the AR room was given the same set of tasks: to start in the bed (a physical bed located to match the plan), go to the bathroom and sit down on the toilet (a physical toilet located to match the plan location), exit the bathroom and go to the patient chair (a physical chair located to match the plan location), and back to the bed. As panel members conducted each segment of the exercise, they were asked to “think aloud” and provide comments based on their design expertise and perceptions of design features that might affect patient stability. They were not asked to role-play a “frail” or elderly patient. Participants were also challenged to “think outside of the box” if they felt there might be a better solution, even if in conflict with a code or guideline.

**Figure 3:**
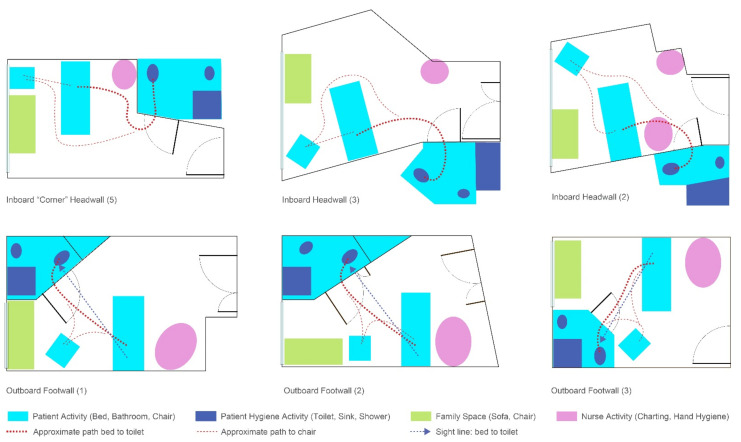
Room configurations used in the AR.

**Figure 4:**
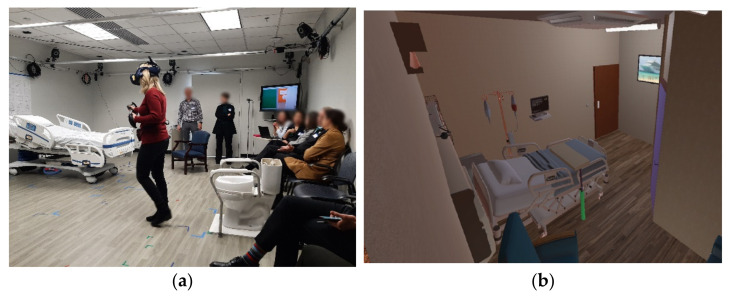
AR activities. (**a**) One AC member completes tasks while others observe them both directly and indirectly (seeing AR participant’s view on a large screen); (**b**) sample view of AR room.

Six of the rooms submitted by the AC were used for AR test “sessions” in which AC members provided feedback while completing or observing tasks in the AR (Figure 3). The AR evaluation activity involved 13 total sessions (two AC members completing tasks for five of the rooms and three AC members completing tasks for a sixth room). Different participants were used in each session. Each AC participant walked through the AR room they had provided, and then at least one other AC member walked through the same room. The remaining AC panel members observed the AR participant and the enactment on a large screen (Figure 4). They offered commentary as the activities progressed.

One AC member indicated a prior issue with motion sickness in VR. This participant was guided in steps to mitigate queasiness by limiting actions such as quickly getting out of the bed or turning quickly to respond to AC comments. The average AR session lasted ten minutes. As each session’s duration was fairly short, none of the participants experienced simulator sickness during the testing. All sessions were recorded (audio and participant’s movements in the AR setting), and one researcher kept detailed notes on participants’ observations and actions while the tasks were being performed.

With an HF/E approach, solutions prioritize fitting the environment to the user rather than training the user to fit the environment [24]. In order to establish user “fit”, comments were coded using a theoretical framework for HF/E design principles and building category permanence [25] to provide a comparative analysis of the different rooms tested.

Due to the small sample size, pre- and post-session Likert-scale survey results were analyzed using the non-parametric Wilcoxon signed-rank test. Means and standard deviations were reported for ease of understanding. A *p*-value < 0.10 was viewed as statistically significant due to the exploratory nature of this study.

## 3. Results

The results include a summary of AC views on the design process and decision-making, AC impressions of the patient rooms simulated in AR, findings of face validity of the heat maps as compared to the AR scenarios, AR and overall debriefs, and pre- and post-session survey results.

### 3.1. AC Views on the Design Process and Decision-Making

The AC panel described the design process as starting with a functional program. This is the critical thinking for a project that starts with asking, “What are you designing for in order for the design to match the operational needs?” The functional program is a “living” document that evolves over the course of design that identifies adjacencies, patient populations, workflow, and patient flows. Patient rooms are designed in zones and while there are typical approaches for inboard and outboard designs, there is no agreement about the best approach. Each healthcare organization may want to set up the room to suit their own preferences within the allowed regulatory requirements. A project team must take into account considerations for safety (e.g., fall prevention, infection prevention); nurse work (e.g., visibility into the room, location of supplies); and the individual patient considerations (e.g., experience, satisfaction). The resulting decisions get incorporated into the room design. Larger organizations may enlist the use of standardized “best practice” templates for design, but even then, there is an iterative process that includes users to inform the project-specific design. These templates are continually updated based on operational feedback and clinician panels.

Consensus building is important, as there are always differences of opinion about priorities. The panel agreed that flexibility is important to accommodate different operations and staff. Feedback from those using a facility is often hampered by the workarounds that have developed (i.e., the ways in which users have adjusted behavior to overcome a barrier to work). Unfortunately, there is little science behind the decision-making, and teams often fall back on intuition or traditional “best practice” approaches when it comes to safety. The best designers will try to distill and balance the thousands of decisions into priorities gathered from the various user groups. However, users come in with preconceived ideas, and the priorities may be skewed by the personality, influence, or “loudness” of individuals in the room. The process was defined by one AC member as “80% function and 20% culture.”

With respect to where falls fit into the priorities, all agreed it was not first on the list. Infection prevention was articulated as a top safety priority, followed by medication safety. Anti-ligature issues have recently become a higher priority than in previous years in the US, and as a result, falls may be the fourth highest priority. Safety also needs to be balanced with the patient and family experience. Surface selection is a continual discussion, especially as to whether materials are durable from a cleaning perspective. From a safety perspective, there are differing views on the level of independence the patient should have in the room. Patients are often encouraged to call for help when toileting, so debates ensue about whether making the toilet visible to the patient encourages them to try to ambulate on their own. This is countered by current trends to promote mobility. Bathroom doors are also a feature of discussion—sliding or swinging, left open or closed. The need for a bathroom door was raised, and the rationale was multi-faceted, including promoting patient dignity, maintaining negative pressure (odors, infection prevention), and providing privacy for family members using the toilet. An example was offered where implementing a sliding door (that might improve safety in the ergonomics of opening the door) would require additional square footage and cost (estimated as an additional USD 2M over the entire project). Teams may try to push the envelope, but without being able to define the cost–benefit, they have minimal power when it comes to the budget.

### 3.2. AC Impressions of the AR Patient Rooms

As summarized in Table 2, Table 3, Table 4, Table 5, Table 6 and Table 7, comments gathered during AR sessions were categorized using a theoretical framework of categorizing for both HF/E design principles and building design permanence [6,25]. Ordered by prevalence in the current study, the design principles include:

- Minimize the need for strength (St): most often associated with muscles in the arm, leg, or back and can be dynamic (e.g., lifting) or static (e.g., holding, gripping). This applies to room/bathroom configuration, toilet location in the bathroom, and the use of grab bars to support weaker patients (e.g., grab bars close enough to reach).
- Optimize movement (Mv): associated with healthy/neutral postures (positioning of bed height, toilet, sink, grab bars, etc.), removing tripping hazards (clutter obstructions), recognizing necessary movement aids (walkers), and facilitating suitable reach and turning.
- Optimize decision-making (Dm): choice may be associated with uncertainty with no clear best option. Mental models help to organize the execution of a task, and task visibility is important in creating a mental model. Minimize the number of components and related tools (equipment) and collocate sequential work elements with the respective tools (equipment) needed to complete each task.
- Minimize manipulation (Ma): prevent awkward positions (i.e., heights, reach, grip, clearances). Parts or equipment should be easy to move, and easy to grip/grasp.
- Minimize perception time (Pe): hidden or invisible parts (e.g., objects, furniture, fixtures) are sometimes forgotten (e.g., a nurse may not be able to visualize a patient from the hallway because of bed location). Visual and tactile discrimination may enhance stimulus-response for reduced reaction time.

### 3.3. Face Validity of Heat Map Models from the AR Experience

An open discussion was held to review the AR experience and whether the observations of the day influenced any perceptions about risk represented in the heat map model. This was conceived as the opportunity to check face validity. In general, the heat maps reflected many of the AC experiences while completing tasks in AR, and there were several specific areas discussed by the AC. There were also limitations noted by the AC.

#### 3.3.1. Heat Maps and Experiential Agreement

The heat maps suggested that room configurations with pull-down grab bars on either side of the toilet offered more stability that a standard ADA-style configuration (wall-mounted on the back wall and one side). The AC panel consistently stated during the AR sessions that the grab bars on either side of the toilet offered a better feeling of safety, although in the AR, these were real bars that were in the down position and did not require manipulation. There were comments suggesting that there are fewer falls in rooms with dual-bar systems. There was also a general consensus that longer unsupported distances, especially as might be required in a larger ADA-sized bathroom, might create higher risk. Just as a smaller bathroom size was discussed for “safer” unsupported ambulation, the tradeoff is a lack of space for assisted toileting, which is often preferred.

Some feedback focused on turning and how much turning was required to get from the bed to positioning one’s self on the toilet, or maneuvering from the toilet to the sink. There was considerable variation between the room designs, and the panelists had a better appreciation for specific fixture orientations in the design. For some, the AR activities challenged their initial assumptions. Questions arose about how many unsupported steps could be taken, especially from the bed to the bathroom door. According to the current model, two to three steps can be taken before the risk increases. The algorithm captures risk associated with distances and turning.

The heat maps suggested room configurations with the bathroom on the headwall may contribute to higher risk due to wall-mounted computers or other equipment that might obstruct the supportive path to the bathroom. Following the sessions, the panel members expressed their views that formal support systems (e.g., grab bars located along the headwall) were often blocked or unused, suggesting some aspects of the modeling were correct.

#### 3.3.2. Heat Map Limitations

After completing the AR tasks, the AC noted the heat maps do not account for risk in assisted versus unassisted activities, as well as individuals of size and the use of patient lifts. The risk models also did not account for stability differences between a mobile workstation as compared to a wall-mounted computer, which could influence the results. The “lower” risk for footwall configurations was seen as lacking the input for patient intrinsic conditions.

The panel members expressed surprise that the swing-style door was not represented as a higher risk than a sliding door. Unfortunately, there are not empirical data to model this variation in the algorithm. The researchers also explained that the room configuration with the sliding door was larger than the available space to conduct the AR sessions. The modelling of risk relating to doors was also difficult to gauge, as there were technical issues with the programming of door manipulation during the AR sessions.

Several AC panelists offered opinions that the IV poles and overbed tables became de facto walkers, but they could also become tripping hazards. There was extensive conversation about the flooring, and currently, the algorithm does not capture the level of detail being discussed relative to specific flooring types and floors in bathrooms being wet or dry (e.g., based on curb or curbless shower designs).

### 3.4. Debrief of the Day’s Activities

During the final debrief discussion of the day’s activities, the group discussed their new insights and interest in continued support of the project. Together with the research team, the AC members discussed the need for empirical data to confirm what the model is predicting. While AC members initially sought to request organizational fall data from the risk management and quality and safety departments, they have since been unable to garner an internal champion willing to share fall-related data for the study.

AC members commented that budgets are established using rules of thumb during master planning and do not consider the life-cycle cost avoidance associated with falls. As a result, when decisions about certain features are raised during design, the inclusion of those features may not be possible due to first-cost financial constraints. Evidence to support features based on ROI is needed to support the added cost. Life-cycle costs of safety are becoming more important, but there are multiple factors driving costs, and this includes aspects of the patient experience. The pay for performance reimbursement model in the US has started to shift thinking to cost-avoidance associated with measured outcomes, namely both safety (e.g., infection prevention) and experiential outcomes (HCAHPS). The panel agreed they are consistently evaluating implications for a building that will be in place for 20–30 years, or more.

Some panelists felt that the evaluation activity brought up more questions than answers regarding commonly accepted design strategies, some of which proved to be potentially problematic for patient safety and stability. For example, prior to the AR evaluation, a headwall bathroom seemed preferable because of the short distance, but post-evaluation it became clear to the AC panel that it is more complicated as the distance must be considered alongside the bathroom configuration and toilet location. The relationship of the parts, relative to the task, can create unintended latent safety conditions. For example, a bathroom door location on the headwall may seem appropriate, as might a bathroom configuration with a straightforward relationship between the toilet and sink. The relationship of the two, however, requires a patient to pivot and turn based on the toilet location and orientation, which may not be adequately considered. The participatory exercise of moving from one destination to another while focusing solely on fall risk changed the perceptions and assumptions.

One of the principal investigators shared next steps with the AC members, including plans to take the data from the day’s session and model new patient room environments for further testing, and ultimately, to develop an optimal room design using an expanded predictive model [14]. While AC members agreed that optimal design considerations would be helpful for the industry, they shared concerns that “an” optimal room design could be interpreted by healthcare owners as a singular explicit solution for safety. This might stifle design innovation and inadequately reflect the various conditions found in healthcare built environments (e.g., structural grid), as well as the competing priorities that vary from organization to organization. The researchers clarified that the “optimal room” findings could be presented as considerations around relationships among features in the room and how they relate to fall risk.

### 3.5. Survey Results: Pre and Post

As shown in Table 8, few of the items posed in the Likert-scale survey changed with respect to statistical significance. The scores were used to order the design considerations from what was perceived as more to less risky. Although most scores tended to trend higher after the day’s sessions, there were changes in where the consideration fell once ranked according to score. There were three exceptions. Visibility from the bed to both the bathroom door and the toilet was perceived to be safer following the AR sessions (*p* = 0.06), as was space on the opening side of the bathroom door (*p* = 0.08).

The results for additional questions about bathroom location and distances are provided in Table 9. While few perceptions were changed about the location of the bathroom, the door type (overwhelmingly sliding doors into the bathroom), or the door widths, there were reductions in the perceived acceptable distances between the bed and bathroom door and unsupported walking distances.

## 4. Discussion

In this study, the focus was on gathering feedback from experts in healthcare inpatient room design, as one stakeholder constituent. Across all designs, many comments centered on design issues related to optimizing movement (i.e., the number of turns, degree of turning) and the need for strength (i.e., the relative distances between destinations and the support devices available). The architects’ perceptions about difficulties with turning are supported by prior research [7,8], although direct participation in experiencing patient paths of movement created a stronger, more visceral reaction to the design conditions necessitating turns and turning. The AC observations focused not only on the relationship of the bed to the bathroom door, but the specific location and orientation of the toilet in the patient bathroom. Toilet orientations that allowed a “side slide” approach from the door (less than 90-degree turn) were seen being as potentially easier to navigate than locations requiring a full turn to sit down. Rather than view design for the patient room and bathroom as independent components in the design exercise, they need to be viewed as a system.

Other comments that emerged over the course of the day coded as movement suggested a possible preference for the bathroom to be on the outboard side—away from the nurse’s work. Two outboard footwall configurations were appreciated for their convenient “triangular” relationship between the bed, the toilet, and the patient room chair. However, the outboard location sets out the potential need to balance competing priorities around nurse work, environmental services (EVS) work, and patient work to access the bathroom. Conversations between AC members included questions about whether you want EVS to cross the room to clean the bathroom and whether you want to make it easier for the patient to ambulate unassisted. For the latter, patients are told “call, don’t fall”, but the reality is that many patients continue to attempt their own activities without assistance [26]. Training, education, and alarms do not provide effective solutions [27], and the environment should be seen as a crucial tool in creating optimal conditions for safety, whether the patient is alone or assisted.

Within the strength category, there was discussion about necessary support. In some configurations, comments were more focused on distances that would necessitate more or less support, and in others the discussion was focused on assistive devices (e.g., grab bars). Grab bars on both sides of the toilet were preferred, and vertical grab bars at bathroom doors were mentioned to support stability at the door in several conditions. It was also noted that vertical grab bars would be susceptible to damage by being knocked. The AC observations surrounding grab bars is also supported by prior studies [28,29,30].

To minimize manipulation, most participants perceived that a sliding door would be preferred, although sliding doors were not available in the AR modelling. There is currently no research to support or refute such assumptions.

While there were similarities among the submitted room designs and the room designs that were tested (the workspace envelope), there were variations in the details of the layout, as well as room sizes. The width and the depth of the room could easily influence both physical travel distances and perceived effort. There was a fine line between a room that felt “safe” with respect to its size and configuration and one that felt too daunting or confined and cluttered. For the more immediate space around the patient (personal workspace), there was an observed tradeoff between what might be beneficial for the unassisted patient (e.g., a closer distance between walls at the toilet) and what would best support assisted activities (e.g., adequate clearance for a nurse). Finally, at the more detailed level of products, the use of dual grab bars (typically including at least one swing-up style) was seen as an effective solution to support stability. This would most often add a second grab bar at the toilet, increasing first costs.

Other patient safety priorities, some of which may take precedence over patient falls, include infection control, medication safety, and behavioral health. With respect to falls, prior to the day’s session, AC panel members were often uncertain about whether the headwall or the footwall was a preferred location for the bathroom. Anecdotally, each could share the “thinking” behind a headwall design (e.g., shorter distances, support on the headwall) but struggled with a lack of evidence to support the decision. Post-evaluation, it became clear that the location of the bathroom in a headwall configuration matters. In rooms where patients must pivot and turn 180 degrees or more degrees to sit on the toilet, there were more concerns about safety and stability. As such, there could be cases where a footwall design, with an appropriate toilet location, is preferred.

There are limitations to this study. While the six healthcare design experts were selected based on their unique abilities to represent a wide breadth and depth of architectural and design experience in the field, this is a small sample size, limiting generalizability. However, each AC member is a recognized leader in the field, and the larger goal in collecting expert feedback is to guide further study, not provide conclusive solutions. As part of an iterative process, the results of the planned human subject study will also inform the development of the algorithm and room design.

The AR model was still in development at the time of the AC meeting, so there were a limited number of room types that could be included and limitations with how specific design features could be represented. For example, the full bathroom was not modeled, the virtual IV pole did not adequately represent the challenges posed by manipulating a physical IV pole, and the door properties in AR often resulted in glitches in opening and closing the door. Additionally, while lighting conditions are an important factor in mitigating the risk of patient falls, the AR model used daylight conditions and did not incorporate detailed variations in lighting types and locations. The risk algorithm [15] incorporates additional detail about lighting types (e.g., overhead, night lights at the bathroom door).

While the study did not include patient or staff feedback, future phases of the study will incorporate both qualitative and quantitative study of the proposed “ideal” room design from all stakeholder groups, including the AC. Finally, while questions arose about the use of the IV pole and overbed table as supportive devices or obstacles, the project is focused on designing the built environment and not designing furniture and/or equipment.

## 5. Conclusions

The environment is a powerful (but often overlooked) tool in creating optimal conditions for safety, whether the patient is ambulating alone or assisted. This was confirmed by the influence of the actual experience of moving within the room designs (through use of AR) on the architects’ perspectives and understanding regarding the safety of the particular room designs. The study supports the benefit of a proactive participatory approach during design, inclusive of all stakeholders (e.g., patients, family staff, design team members), even though the architects and designers are often seen as the experts who are engaged to distill the information about user feedback. This study, focused solely on the professional feedback from the AC, found subtle shifts in perceptions regarding the efficacy of design solutions through active participation in the AR scenarios of taking the path of a patient. In the short-term, the findings from AC feedback will be used to inform computerized algorithms for both evaluating risk and designing the “optimal” patient room for stability. In the long term, the study will enable architects through empirical data-driven results that, when implemented, will have the potential for mitigating the risk of falls, reducing patient harm, and potentially saving lives, as well as reducing costs to the hospital.

## Figures and Tables

**Table 1:** National Design Advisory Council affiliations.

Participant	Healthcare Organization	Regulatory/ Advisory Role	Association KC Leadership	Architect	Professional Certification
1	large	x		x	x
2				x	x
3	medium			*	x
4		x		x	x
5	small	x	x	x	x
6		x			x
7		x		x	x

* Trained as an architect, but not licensed.

**Table 2:** Session 1–2 feedback (Room 1—inboard corner headwall).

Session and Room Configuration	Built Environment Permanence	Strength (St)	Movement (Mv)	Decision-Making (Dm)	Manipulation (Ma)	Perception (Pe)
Sessions 1–2 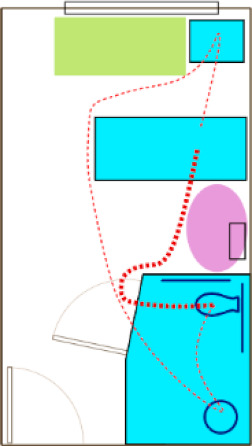	Workspace Envelope	The bathroom door is near the bed; a smaller bathroom would reduce distances from toilet to sink	The bathroom location is a straight path to the door—not much turning required; never enough horizontal surfaces for nurses—they use the over-bed table so it is never in the same place and becomes an obstruction (clutter)	You can see the toilet door (if open) from the bed	Split doors at the bathroom might be easier to use; auto-open door would be ideal, but there can be challenges (e.g., Jewish Orthodox power on Sabbath)	Distance from the bathroom to the chair seems long
	Personal Workspace		You have to reach over the toilet to use the ADA-compliant grab bars			
	Products	A grab bar on the wall, opposite the bed, would aid stability; grab bar in the bathroom offers some support—double grab bars at the toilet would be better for support; continuous rails or shelf to the sink (and at the front of the sink) might offer more support; bedrail at the foot of the bed was used for support walking to chair				Because the over-bed table is mobile, it was not used for support

**Table 3:** Session 3–4 Feedback (Room 2—Inboard Headwall).

Session and Room Configuration	Built Environment Permanence	Strength (St)	Movement (Mv)	Decision-Making (Dm)	Manipulation (Ma)	Perception (Pe)
Sessions 3–4 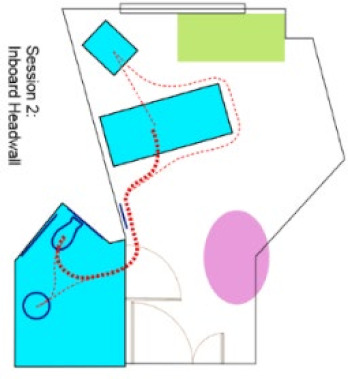	Workspace Envelope	Room has lots of angles; bathroom on footwall might be closer but creates unsupported walking	Lots of turning and torquing; toilet placement wrong—too much turning; a short triangle of bed/bathroom/ chair might work	Bathroom door visible once you get out of bed; do not see the toilet	No clearance for staff assistance at the patient chair	Nurse can see patient head; bathroom feels far away from bed; chair seems far from bathroom
	Personal Workspace	Sink in front of/close to toilet				Chair looks wedged in by sofa—a disincentive to use? (feels like more space in AR than shown in plan)
	Products	Bed rail and wall rail for fairly continuous support; maybe need an additional wall grab bar in bathroom for support to toilet; double bars at toilet would be better—rail on far side; rail at toilet provides support to sink, but no support sink to door; hold bed to get to chair—not good support	Over-bed table in the way; mobile workstation (WOW), not in model, could be an obstruction at the bed (not sure whether WOW gets moved back to proper spot)		Manipulation of door with IV can be difficult	

**Table 4:** Session 5–7 Feedback (Room 3—Inboard Headwall).

Session and Room Configuration	Built Environment Permanence	Strength (St)	Movement (Mv)	Decision-Making (Dm)	Manipulation (Ma)	Perception (Pe)
Sessions 5–7 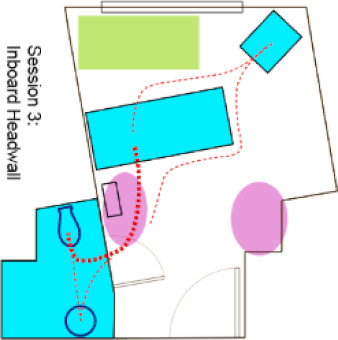	Workspace Envelope	Shorter walk to toilet; chair at footwall corner closer to toilet than other layout (decision to move chair from sofa corner for better distance / mobility); chair better at footwall; room seems smaller; generous space at chair, but patient and family cannot see TV. No support to the bathroom door—use IV pole/ headwall ledge for support	Wall-mounted monitor would be in the way; have to do a 180 degree turn for toilet and back up, but less torquing; it “looked” better when someone else was getting to toilet, but it is not; potential conflicts with work flow if staff use footwall area; straight path to bed from chair, but over-bed table in the way; maybe bathroom should be on family zone side verses nurse work space	Bathroom layout makes it more visible than Session 2	Manipulate door, but pretty easy if door pulls out; no room at toilet for nurse to assist—narrow	Side chair looks like an obstacle; feels tight; I see an obstacle course—room cluttered with furniture
	Personal Workspace	Walls on either side of toilet can be used as a brace, feels safer, but bars on both sides of toilet would be better				
	Products	Want to use bed opposite chair for support (sit/stand)				

**Table 5:** Session 8–9 Feedback (Room 4—Outboard Footwall).

Session and Room Configuration	Built Environment Permanence	Strength (St)	Movement (Mv)	Decision-Making (Dm)	Manipulation (Ma)	Perception (Pe)
Sessions 8–9 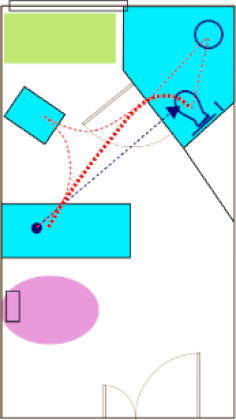	Workspace Envelope	Distances: Very close—1 or 2 steps; the chair is also right there; configuration of patient activity is in a triangle—everything is there	Easy—barely had to turn; moving to chair had no obstacles, but it would depend on the over-bed table location	Can see the bathroom (and toilet if door open); ongoing question—do you want a view of the toilet?	Since the door swings out, it will be closed; sliding door would be better (or door might swing the other way); consider an accordion door; space for nurse to help (in open door)	
	Personal Workspace	Touch the wall next to the door	Side transfer on to toilet; toilet position good; space at toilet good			
	Products	Grab bar at toilet is right there; can use bed for support; possible vertical bar at door for support				

**Table 6:** Session 10–11 Feedback (Room 5—Outboard Footwall).

Session and Room Configuration	Built Environment Permanence	Strength (St)	Movement (Mv)	Decision-Making (Dm)	Manipulation (Ma)	Perception (Pe)
Sessions 10–11 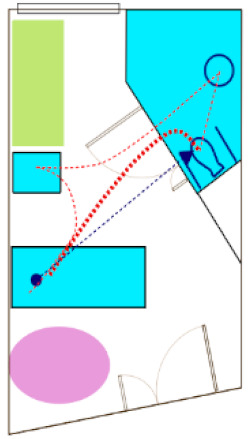	Workspace Envelope	#10: Easy—close, right there; if most falls are bed to toilet, further into the room is an issue feels safe with the wall for support; #11—like bathroom better on headwall—going across seems more daunting	Room to get out of bathroom; do not have to walk around anything to chair—a direct route	If relief panel is closed—would not see toilet	Inboard for nurse-access to patient is better	
	Personal Workspace				Room to stand to pull open door	
	Products	Could put vertical grab bar at door				

**Table 7:** Session 12–13 Feedback (Room 6—Outboard Footwall).

Session and Room Configuration	Built Environment Permanence	Strength (St)	Movement (Mv)	Decision-Making (Dm)	Manipulation (Ma)	Perception (Pe)
Sessions 12–13 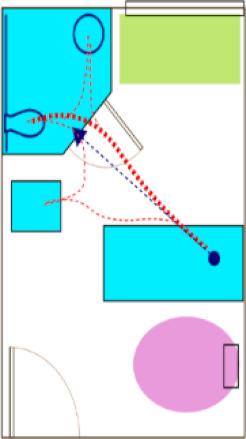	Workspace Envelope	Path to toilet felt long, but path to patient chair easy; room feels smaller and while closer proximity is nice, it feels tight; proximity of chair to toilet awkward—could move	More maneuvering and turning in toilet area—turning 180 degrees not as good vs. side slide; easy transition to chair next to bathroom door	Everything is visible from the bed	Tight at door to bathroom with an IV pole	Nurse can see the patient chair
	Personal Workspace					
	Products					

**Table 8:** Likert-scale survey results, pre- and post-session.

Design Considerations and Perceived Risk ^1^	Mean (SD)		Δ	*p*-Value	Rank	
	Pre	Post			Pre Session	Post Session
Grab bars on both sides of the toilet	4.71 (0.49)	5.00 (0.00)	0.29↑	0.32	2	1
Flush transitions in walking surfaces or between floor types	4.86 (0.38)	5.00 (0.00)	0.14↑	0.32	1	1
An unobstructed path to bathroom (e.g., clutter, equipment, furniture)	4.00 (1.53)	4.67 (0.82)	0.67↑	0.18	10	3
Grab bars/rails on the wall to support walking to the bathroom	4.43 (0.79)	4.67 (0.52)	0.24↑	0.32	3	3
Places to put personal items in reach (e.g., a charging cell phone/tablet)	4.43 (0.53)	4.67 (0.82)	0.24↑	0.56	3	3
A night light fixture near the floor in the pathway toward the patient toilet room	4.71 (0.49)	4.50 (0.84)	−0.21↓	0.32	2	6
Visibility from the bed to the bathroom door	3.14 (1.07)	4.33 (0.82)	1.19↑	0.06 *	16	7
Space on the door opening side of the bathroom door	3.83 (0.41)	4.33 (0.82)	0.50↑	0.08 *	13	7
Flooring patterns that minimize contrast	4.14 (0.38)	4.33 (0.52)	0.19↑	0.56	9	7
Grab bars to support moving from the toilet to the sink	4.29 (0.95)	4.33 (0.82)	0.05↑	1.00	5	7
Flooring materials that minimize glare	4.29 (0.76)	4.33 (0.82)	0.05↑	0.56	5	7
Call bells/pull strings in reach of where falls happen often	4.29 (0.76)	4.00 (1.10)	−0.29↓	0.70	5	13
Defined zones for patient/ family/ clinical activities	3.86 (0.69)	3.83 (0.41)	−0.02↓	0.56	11	14
Contrast between floors and walls	3.86 (0.69)	3.83 (0.75)	−0.02↓	0.32	11	14
Visibility from the bed to the toilet fixture	2.86 (1.07)	3.67 (0.82)	0.81↑	0.06 *	17	15
The ability for the nurse to see the patient’s head during regular rounding	3.43 (0.53)	3.33 (0.52)	−0.10↓	1.00	15	16
Grab bars meeting the ADA minimum at the toilet	3.57 (0.98)	2.67 (1.21)	−0.90↓	0.18	14	17

^1^ 5-point Likert scale—1: introduces much more risk; 3: no more/no less risk; 5: Introduces much less risk. * significant *p*-value < 0.10.

**Table 9:** Perceptions about room design features and distances.

Participant	Survey Round	The Ideal Room Would Have the Bathroom Located on the:	The Ideal Room Would Have the Bathroom:	The Ideal Opening to the Patient Bathroom Has:	Ideal Distance to the Bathroom Door is (x′-y″)	Maximum Unsupported Patient Walking Distance (x′-y″)	Ideal Clear Door Width to the Bathroom (x′-y″)	Additional Capital Costs Are Justifiable in the Context of Cost Avoidance from Falls: (5-Point Likert scale, 1 = Strongly Agree)
P1	Pre	HW	IB	Door/relief panel	6′	2′	36″	2
	Post	HW	IB	Single	5′	2′	36″	2
P2	Pre	FW	OB	Sliding	4–5′	4′	48″	1
	Post	FW	OB	Sliding	3–4′	3′	42″	1
P3	Pre	HW	IB	Sliding	4′	3′		2
	Post	FW	IB	Sliding or door/relief	<4′	<3′	48″	1
P4	Pre	NS	IB	Sliding	4′	1.5′	42″	2
	Post	NS	IB	Sliding	3′ FW, 3′ + HW	<3′	42″	2
P5	Pre	NS	NS	Sliding	4′	1.5′	42″	1
	Post	NS	NS	Sliding	3.5′	1–1.5′	42″	1
P6	Pre	NS	NS	Sliding	NS	NS	44″	1
	Post	FW	NS	Sliding	5′	5′	44″	1

Legend: HW: headwall; FW: footwall; IB: inboard; OB: outboard, NS: not sure.

## Data Availability

The datasets generated during and/or analysed during the current study are not publicly available due to privacy of the participants but are available from the corresponding author on reasonable request.

## Supplementary Materials

The following are available online at https://www.mdpi.com/article/10.3390/ijerph18168769/s1, Table S1: Patient Room Design Risk Estimation Survey.

## References

[1] National Quality Forum (NQF) (2011). Serious Reportable Events in Healthcare—2011 Update: A Consensus Report.

[2] Centers for Medicare and Medicaid Services (CMS) (2013). Medicare Program. Hospital Inpatient Prospective Payment Systems for Acute Care Hospitals and the Long-Term Care Hospital Prospective Payment System and Fiscal Year 2014 Rates; Quality Reporting Requirements for Specific Providers; Hospital Conditions of Participation; Payment Policies Related to Patient Status. Final Rules.

[3] Centers for Medicare and Medicaid Services (CMS) (2021). Hospital-Acquired Condition Reduction Program (HACRP) Overview. https://qualitynet.cms.gov/inpatient/hac.

[4] Centers for Medicare and Medicaid Services (CMS) (2021). Hospital-Acquired Conditions (Present on Admission Indicator). https://www.cms.gov/Medicare/Medicare-Fee-for-Service-Payment/HospitalAcqCond.

[5] Agency for Healthcare Research and Quality (AHRQ) (2020). AHRQ National Scorecard on Hospital-Acquired Conditions: Final Results for 2014 Through 2017.

[6] Taylor E., Hignett S. (2016). The SCOPE of hospital falls. HERD Health Environ. Res. Des. J..

[7] Pati D., Valipoor S., Cloutier A., Yang J., Freier P., Harvey T.E., Lee J. (2017). Physical design factors contributing to patient falls. J. Patient Saf..

[8] Pati D., Lee J., Mihandoust S., Zadeh M.K., Oh Y. (2018). Top five physical design factors contributing to fall initiation. HERD Health Environ. Res. Des. J..

[9] Taylor D., Merryweather A., Morse J., Wong B. The natural sit-to-stand-walk of the frail. Proceedings of the ASME 2019 International Mechanical Engineering Congress and Exposition.

[10] Xu H., Li X., Shi Y., An L., Taylor D., Christman M., Morse J., Merryweather A. (2020). Hospital bed height influences biomechanics during bed egress: A comparative controlled study of patients with Parkinson disease. J. Biomech..

[11] Cloutier A., Yang J., Pati D., Valipoor S. (2016). Experimental identification of potential falls in older adult hospital patients. J. Biomech..

[12] Mackey D.C., Lachance C.C., Wang P.T., Feldman F., Laing A.C., Leung P.M., Hu X.J., Robinovitch S.N. (2019). The flooring for injury prevention (FLIP) study of compliant flooring for the prevention of fall-related injuries in long-term care: A randomized trial. PLoS Med..

[13] Drahota A.K., Ward D., Udell J., Soilemezi D., Ogollah R., Higgins B., Dean T., Severs M. (2013). Pilot cluster randomised controlled trial of flooring to reduce injuries from falls in wards for older people. Age Ageing.

[14] Chaeibakhsh S., Novin R., Hermans T., Merryweather A., Kuntz A. Optimizing hospital room layout to reduce the risk of patient falls. Proceedings of the 10th International Conference on Operations Research and Enterprise Systems, Online Streaming.

[15] Novin R.S., Taylor E., Hermans T., Merryweather A. (2020). Development of a novel computational model for evaluating fall risk in patient room design. HERD Health Environ. Res. Des. J..

[16] Patterson E.S., Sanders E.B.-N., Sommerich C.M., Lavender S.A., Li J., Evans K.D. (2017). Meeting patient expectations during hospitalization: A grounded theoretical analysis of patient-centered room elements. HERD Health Environ. Res. Des. J..

[17] Lavender S.A., Sommerich C.M., Sanders E.B.-N., Evans K.D., Li J., Umar R.Z.R., Patterson E.S. (2019). Developing evidence-based design guidelines for medical/surgical hospital patient rooms that meet the needs of staff, patients, and visitors. HERD Health Environ. Res. Des. J..

[18] Patterson E.S., Sanders E., Sommerich C.M., Evans K.D., Lavender S.A., Li J. The environmental services perspective on hospital room design: A mixed-methods approach. Proceedings of the 2017 HFES International Symposium on Human Factors and Ergonomics in Health Care.

[19] Lavender S.A., Sommerich C.M., Patterson E., Sanders E.B.-N., Evans K.D., Park S., Umar R.Z.R., Li J. (2015). Hospital patient room design. HERD Health Environ. Res. Des. J..

[20] Gandhi R.D., Patel D.S. (2018). Virtual reality—Opportunities and challenges. Int. Res. J. Eng. Technol..

[21] Milovanovic J., Moreau G., Siret D., Miguet F. Virtual and augmented reality in architectural design and education: An immersive multimodal platform to support architectural pedagogy. Proceedings of the 17th International Conference, CAAD Futures 2017.

[22] Freitas M., Ruschel R. What is happening to virtual and augmented reality applied to architecture?. Proceedings of the 18th International Conference on Computer-Aided Architectural Design Research in Asia.

[23] Sargent R.G. (2013). Verification and validation of simulation models. J. Simul..

[24] Dul J., Bruder R., Buckle P., Carayon P., Falzon P., Marras W.S., Wilson J.R., Van Der Doelen B. (2012). A strategy for human factors/ergonomics: Developing the discipline and profession. Ergonomics.

[25] Taylor E., Hignett S. (2021). Deep scope: A framework for safe healthcare design. Int. J. Environ. Res. Public Health.

[26] Haines T.P., Day L., Hill K.D., Clemson L., Ao C.F. (2014). “Better for others than for me”: A belief that should shape our efforts to promote participation in falls prevention strategies. Arch. Gerontol. Geriatr..

[27] Barker A.L., Morello R.T., Wolfe R., Brand C.A., Haines T., Hill K., Brauer S., Botti M., Cumming R., Livingston P.M. (2016). 6-pack programme to decrease fall injuries in acute hospitals: Cluster randomised controlled trial. BMJ.

[28] Lee S.J., Sanford J., Calkins M., Melgen S., Endicott S., Phillips A. (2017). Beyond ADA accessibility requirements: Meeting seniors’ needs for toilet transfers. HERD Health Environ. Res. Des. J..

[29] Sanford J., Bosch S.J. (2013). An investigation of noncompliant toilet room designs for assisted toileting. HERD Health Environ. Res. Des. J..

[30] Lee S.J., Mehta-Desai R., Oh K., Sanford J., Prilutsky B.I. (2018). Effects of bilateral swing-away grab bars on the biomechanics of stand-to-sit and sit-to-stand toilet transfers. Disabil. Rehabil. Assist. Technol..

