# Supplementary material for: Designing a Patient Room as a Fall Protection Strategy: The Perspectives of Healthcare Design Experts
Source: Int J Environ Res Public Health. 2021 Aug 19;18(16):8769. doi: 10.3390/ijerph18168769 (PMC8392568; doi:10.3390/ijerph18168769)
Supplement: Supplementary file 1 [file ijerph-18-08769-s001.zip › ijerph-1300842-supplementary.pdf]

Patient Room Design Risk Estimation Survey

Consider a med-surg room, often used by those older than 65. Many design considerations may directly or indirectly influence the stability of a patient ambulating in a patient room. Based on your expert opinion, please evaluate the following design considerations relative to introducing the risk of falling in the patient room. There may be items that are difficult to determine because “it depends” – on a variety of potential factors. In these cases, please rate on what you feel is the most common scenario.

If you have comments or clarifications, please hold them for one of our discussions today.

Which elements introduce more or less risk in a patient room/ bathroom designed to support patient stability to mitigate the risk of falls?

	1) Much more risk	*2) More risk*	*3) No more/ no less risk*	*4) Less risk*	*5) Much less risk*
1) The ability for the nurse to see the patient's head during regular rounding	❍	❍	❍	❍	❍
2) Defined zones for patient/ family/ caregiver activities	❍	❍	❍	❍	❍
3) Visibility from the bed to the bathroom door	❍	❍	❍	❍	❍
4) Visibility from the bed to the toilet fixture	❍	❍	❍	❍	❍
5) A night light fixture near the floor in the pathway toward the patient toilet room	❍	❍	❍	❍	❍
6) Grab bars/rails on the wall to support patients walking to the bathroom	❍	❍	❍	❍	❍
7) Grab bars meeting the ADA minimum at the toilet	❍	❍	❍	❍	❍
8) Grab bars on both sides of the toilet	❍	❍	❍	❍	❍
9) Grab bars to support patients moving from the toilet to the sink	❍	❍	❍	❍	❍
10) An unobstructed path to bathroom (e.g., clutter, equipment, furniture)	❍	❍	❍	❍	❍

	1) Much more risk	***2) More risk***	***3) No more/ no less risk***	***4) Less risk***	***5) Much less risk***
11) Space on the door opening side of the bathroom door	❍	❍	❍	❍	❍
12) Flush transitions in walking surfaces or between flooring types	❍	❍	❍	❍	❍
13) Flooring materials that minimize glare	❍	❍	❍	❍	❍
14) Flooring patterns that minimize contrast	❍	❍	❍	❍	❍
15) Contrast between floors and walls	❍	❍	❍	❍	❍
16) Places to put personal items in reach (e.g., a charging cell phone/tablet)	❍	❍	❍	❍	❍
17) Call bells/pull strings in reach of where falls happen most often.	❍	❍	❍	❍	❍

Just focusing on falls… To mitigate the risk of falls by optimizing patient stability:

1) The ideal room would have the bathroom on the (circle one): *Headwall* *Footwall* *Not sure*

2) The ideal room would have the bathroom (circle one): *Inboard* *Outboard* *Not sure*

3) The ideal opening to the patient bathroom has (circle one): *No door* *A single door* *A standard door w/a relief panel (8-12”)* *A door and a half* *A double door (equal leaf panels)* *A sliding door*

4) An ideal distance to the bathroom door is (x’-y”) ________________________

5) The maximum unsupported distance a patient would walk *should be* (x’-y”) ________________________

6) An ideal clear door width to the bathroom would be (x’-y”) ________________________

7) Additional capital costs are justifiable in the context of cost avoidance from falls (circle one): *1: Strongly Agree* *2: Agree* *3: Neutral* *4: Disagree* *5: Strongly Disagree*

Name:____
